# Feasibility of Regional Lymphadenectomy for Stomach-Preserving Surgery in Early Gastric Cancer Omitting Sentinel Node Navigation: A Post Hoc Analysis of the SENORITA Trial

**DOI:** 10.1245/s10434-024-15950-1

**Published:** 2024-07-31

**Authors:** Sin Hye Park, Young-Woo Kim, Jae-Seok Min, Hong Man Yoon, Ji Yeong An, Bang Wool Eom, Hoon Hur, Young Joon Lee, Gyu Seok Cho, Young-Kyu Park, Mi Ran Jung, Ji-Ho Park, Woo Jin Hyung, Sang-Ho Jeong, Myeong-Cherl Kook, Mira Han, Byung-Ho Nam, Keun Won Ryu

**Affiliations:** 1 Center of Gastric Cancer, National Cancer Center, Goyang, Republic of Korea; 2 Present Address: Department of Surgery, Eunpyeong St. Mary’s Hospital, College of Medicine, The Catholic University of Korea, Seoul, Republic of Korea; 3 Department of Surgery, Dongnam Institute of Radiological and Medical Sciences, Cancer Center, Busan, Republic of Korea; 4 Present Address: Division of Foregut Surgery, Korea University College of Medicine, Korea University Anam Hospital, Seoul, Republic of Korea; 5 Department of Surgery, Samsung Medical Center, Sungkyunkwan University School of Medicine, Seoul, Republic of Korea; 6 Department of Surgery, Ajou University School of Medicine, Suwon, Republic of Korea; 7 Department of Surgery, Gyeongsang National University, Jinju, Republic of Korea; 8 Department of Surgery, Soonchunhyang University College of Medicine, Bucheon, Republic of Korea; 9 Department of Surgery, Chonnam National University Hwasun Hospital, Hwasun, Republic of Korea; 10 Department of Surgery, Yonsei University College of Medicine, Seoul, Republic of Korea; 11 Biostatistics Collaboration Team, National Cancer Center, Goyang, Republic of Korea; 12 Present Address: Department of Medical Research Collaborating Center, Seoul Metropolitan Government - Seoul National University Boramae Medical Center, Seoul, Republic of Korea; 13 Present Address: Clinical Design Research Center, HERINGS, The Institution of Advanced Clinical and Biomedical Research, Seoul, Republic of Korea

**Keywords:** Stomach neoplasm, Early gastric cancer, Sentinel lymph node, Lymph node metastasis, Lymphadenectomy

## Abstract

**Background:**

Sentinel node navigation (SNN) has been known as the effective treatment for stomach-preserving surgery in early gastric cancer; however, SNN presents several technical difficulties in real practice.

**Objective:**

This study aimed to evaluate the feasibility of regional lymphadenectomy omitting SNN, using the post hoc analysis of a randomized controlled trial.

**Methods:**

Using data from the SENORITA trial that compared laparoscopic standard gastrectomy with lymphadenectomy and laparoscopic SNN, 237 patients who underwent SNN were included in this study. Tumor location was divided into longitudinal and circumferential directions. According to the location of the tumor, the presence or absence of lymph node (LN) metastases between sentinel and non-sentinel basins were analyzed. Proposed regional LN stations were defined as the closest area to the primary tumor. Sensitivities, specificities, positive predictive values, and negative predictive values (NPV) of SNN and regional lymphadenectomy were compared.

**Results:**

Metastasis to non-sentinel basins with tumor-free in sentinel basins was observed in one patient (0.4%). The rate of LN metastasis to non-regional LN stations without regional LN metastasis was 2.5% (6/237). The sensitivity and NPV of SNN were found to be significantly higher than those of regional lymphadenectomy (96.8% vs. 80.6% [*p* = 0.016] and 99.5% vs. 97.2% [*p* = 0.021], respectively).

**Conclusions:**

This study showed that regional lymphadenectomy for stomach-preserving surgery, omitting SNN, was insufficient; therefore, SNN is required in stomach-preserving surgery.

**Supplementary Information:**

The online version contains supplementary material available at 10.1245/s10434-024-15950-1.

Currently, the standard treatment for early gastric cancer (EGC) is endoscopic resection or gastrectomy.^1^ If the tumor does not meet indications for endoscopic resection, gastrectomy with lymphadenectomy can be performed. Although the lymph node (LN) metastasis rate for EGC is approximately 10.0–16.0%,^2–5^ a significant extent of gastrectomy and radical LN dissection can be performed for EGC treatment.

Sentinel node navigation surgery (SNNS) is an alternative procedure introduced to reduce extensive lymphadenectomy and preserve the stomach volume and function, thereby improving the quality of life of patients.^6^ In the recently published SENORITA trial,^7–9^ laparoscopic sentinel basin dissection and stomach-preserving surgery for EGC treatment reported similar overall survival rates and better quality of life compared with laparoscopic standard gastrectomy and lymphadenectomy.

However, SNNS requires injecting a tracer (radioisotope and dye) around the tumor using pre- and/or intraoperative endoscopy, detecting sentinel basins with the naked eye for dye, fluorescence, or radioactivity with special devices, and isolating the nodes.^10^ This surgical process is difficult to generalize when considering technical aspects, operation time, and surgical manpower.

In EGC, LN metastasis tends to be located in the closest LN stations (regional LN) based on the location of the primary tumor, but skip metastases are possible. If regional lymphadenectomy could be performed without the more complicated use of SNN, stomach-preserving surgery could be performed more easily. Moreover, regional lymphadenectomy may reduce the risk of missing skip metastases.

The purpose of this study was to evaluate the feasibility of performing regional lymphadenectomy without SNNS using a post hoc analysis of data from the SENORITA randomized controlled trial.

## Materials and Methods

### Patients

This study obtained data from SENORITA, a multicenter, randomized controlled trial that compared laparoscopic standard gastrectomy with lymphadenectomy and laparoscopic SNNS.^7,11^ Between March 2013 and December 2016, 580 patients with clinical stage T1N0M0 gastric cancer that was < 3 cm in tumor size and located at least 2 cm apart from the pylorus and cardia were included in the SENORITA trial.^10^ The patients were randomly allocated into the laparoscopic standard gastrectomy (269 patients) and laparoscopic SNNS (258 patients) groups after excluding 53 patients. In the laparoscopic SNNS group, sentinel navigation procedures were not possible in 21 patients due to the following reasons: suspected T2 or higher, gross LN metastasis, large tumor, tumor location near the pylorus and cardia, newly detected ulcer lesion, and failure to detect the sentinel basin. Finally, 237 patients underwent laparoscopic SNNS.

This study was performed in accordance with the principles of the 1964 Declaration of Helsinki. Since this study used anonymized data from patients in the previous study, patient consent was waived. This study was approved by the Institutional Review Board of the National Cancer Center (approval number NCC. 2023-0190)

### Proposed Regional Lymphadenectomy According to the Tumor Location

Clinicopathological characteristics of patients who received laparoscopic SNNS were evaluated. Tumor location was categorized into longitudinal (upper, middle, and lower thirds) and circumferential (anterior wall, greater curvature, lesser curvature, and posterior wall) directions.^12^ Pathologic tumor-node-metastasis stage was evaluated according to the International Union Against Cancer/American Joint Committee on Cancer staging system.^13^

The relationship between LN metastases and sentinel and non-sentinel basins according to tumor location was investigated. If the treatment was completed with laparoscopic SNNS due to the absence of LN metastasis in the sentinel basins without recurrence during the follow-up period, these cases were considered negative metastasis in the non-sentinel basins. In contrast, if LN recurrence occurred after laparoscopic SNNS, this was considered metastasis in the non-sentinel basins.

The regional LN area was defined as the peri-gastric nodal station closest to the tumor, considering that nodal metastases were likely to occur (Table 1). We assumed that tumors located on the anterior or posterior side would metastasize to the peri-gastric LNs on both the lesser and greater curvature sides. If LN metastases to the regional LN stations, these were regarded as regional LN metastases, and if LN metastases to stations other than regional LN stations, these were considered non-regional LN metastases. The effectiveness of SNN and regional lymphadenectomy was compared in terms of sensitivity, false negative rate, specificity, positive predictive value (PPV), and negative predictive value (NPV).

**Table 1:** Proposed definition of regional lymph node station based on tumor location

	Upper third	Middle third	Lower third
Lesser curvature	No. 3	No. 3	No. 3
Greater curvature	No. 4sb	No. 4d	No. 4d, 6
Anterior and posterior wall	No. 3, 4sb	No. 3, 4d	No. 3, 4d, 6

### Procedures for Sentinel Basin Dissection

The procedures of sentinel basin dissection have been described in detail in the SENORITA protocol.^10^ Using intraoperative endoscopy, 1 mL each of dual tracers (mixture of indocyanine green and radiolabeled human serum albumin) was injected into four directions of the submucosal layer of the primary tumor. After 15 min from the first injection, the sentinel basins were detected using the naked eye for dye and a gamma probe for radioactivity. Dissected basins were extracted and sentinel basin nodes were harvested in the operating room. These nodes were sent to the pathologist to assess the presence of the tumor using a frozen examination. If sentinel basin nodes were positive in frozen sections, standard gastrectomy and lymphadenectomy should be performed. When sentinel nodes were tumor-free in frozen sections, stomach-preserving surgery could proceed. All sentinel basin nodes and non-sentinel basins were re-evaluated postoperatively.

### Statistical Analysis

Continuous variables were presented as medians and interquartile ranges, and categorical variables as numbers and percentages. The diagnostic performance (sensitivity, specificity, PPV, and NPV) was compared using the bootstrapping method with 1000 resamples and expressed as percentages and 95% confidence intervals. Statistical significance was set at *p* < 0.05. All statistical analyses were conducted using SAS software version 9.4 (SAS Institute, Inc., Cary, NC, USA).

## Results

### Clinicopathologic Outcomes of Patients

Of the 237 patients who underwent SNN, over 60% were male, and middle-third tumors were the most common location (59.5%, 141/237) [Table 2]. Regarding tumor sites in circumferential parts of the stomach, the most common were the lesser and greater curvature sides (33.3% each). The median tumor size was 2.0 cm. Regarding the extent of gastric resection, laparoscopic wedge resection (80.2%, 190/237) was more frequently performed. One sentinel basin was detected in 53.6% of patients. The median number of sentinel nodes was eight. Furthermore, 94.5% of patients had pathologic stage I disease (224/237), and 30 patients had nodal metastasis in the pathologic results.

**Table 2:** Clinicopathological characteristics of patients who underwent sentinel node navigation surgery

Variable	Patients [*n* = 237]
Age, years [median (IQR)]	55.0 (48.0–64.0)
BMI, kg/m^2^ [median (IQR)]	23.0 (22.0–26.0)
Sex	
Male	144 (60.8)
Female	93 (39.2)
Longitudinal location of the tumor	
Upper third	13 (5.5)
Middle third	141 (59.5)
Lower third	83 (35.0)
Circumferential location of the tumor	
Anterior wall	37 (15.6)
Greater curvature	79 (33.3)
Lesser curvature	79 (33.3)
Posterior wall	42 (17.7)
Tumor size, cm (IQR)	1.7 (1.2–2.0)
Histology	
Tubular adenocarcinoma	139 (58.6)
Signet ring cell carcinoma	98 (41.4)
Extent of gastric resection	
LWR	190 (80.2)
LSR	18 (7.6)
ESD	2 (0.8)
LDG	23 (9.7)
LPPG	1 (0.4)
LTG	2 (0.8)
ODG	1 (0.4)
Number of sentinel basins	
1	127 (53.6)
2	89 (37.6)
3	21 (8.9)
Total number of sentinel nodes [median (IQR)]	8.0 (4.0–14.0)
Pathological T classification	
T1a	141 (59.5)
T1b	83 (35.0)
T2	8 (3.4)
T3	5 (2.1)
Pathological N classification	
N0	207 (87.3)
N1	23 (9.7)
N2	4 (1.7)
N3	3 (1.3)
Pathological stage	
Ia	200 (84.4)
Ib	24 (10.1)
IIa	7 (3.0)
IIb	5 (2.1)
IIIb	1 (0.4)

Data are expressed as *n* (%) unless otherwise specified

IQR interquartile range, BMI body mass index, LWR laparoscopic wedge resection, LSR laparoscopic segmental resection, ESD endoscopic submucosal dissection, LDG laparoscopic distal gastrectomy, LPPG laparoscopic pylorus-preserving gastrectomy, LTG laparoscopic total gastrectomy, ODG open distal gastrectomy

### Distribution of Lymph Node Metastases to Sentinel Basins and/or Non-Sentinel Basins According to Tumor Location

The distribution of sentinel basins and LN metastases in each LN station according to tumor locations is presented in electronic supplementary Table 1. The rate of LN metastasis in the sentinel basins and/or non-sentinel basins for each tumor location was 13.1% (Table 3). Nodal metastases in the sentinel basins were observed, except for tumors in the upper third and lesser curvature sites. When the tumors were in the middle third and lesser curvature of the stomach, the rate of metastases in both sentinel and non-sentinel basins was 4.0%. In one patient whose tumor was located in the lower third and greater curvature, and tumor free in the sentinel basins (LN #4d), LN recurrence occurred in the non-sentinel LN (LNs #6, #7, and #11p) during the follow-up period (0.4%, 1/237).

**Table 3:** Distribution of lymph node metastases to sentinel basins and/or non-sentinel basins according to tumor location

	Upper-LC [*n* = 3]	Upper-GC [*n* = 5]	Upper-AP [*n* = 5]	Middle-LC [*n* = 50]	Middle-GC [*n* = 46]	Middle-AP [*n* = 45]	Lower-LC [*n* = 26]	Lower-GC [*n* = 28]	Lower-AP [*n* = 29]
Sentinel basin LNM (−)	3 (100)	4 (80.0)	4 (80.0)	44 (88.0)	39 (84.8)	40 (88.9)	24 (92.3)	23 (82.1)	25 (86.2)
Sentinel basin LNM (+) only	0 (0)	1 (20.0)	1 (20.0)	4 (8.0)	7 (15.2)	5 (11.1)	2 (7.7)	4 (14.3)	4 (13.8)
Sentinel basin and non-sentinel LNM (+)	0 (0)	0 (0)	0 (0)	2 (4.0)	0 (0)	0 (0))	0 (0)	0 (0))	0 (0)
Non-sentinel LNM (+) only	0 (0)	0 (0)	0 (0)	0 (0)	0 (0)	0 (0)	0 (0)	1 (3.6)	0 (0)

Data are expressed as *n* (%)

LC lesser curvature, GC greater curvature AP anterior and posterior, LNM lymph node metastasis

### Distribution of Lymph Node Metastases to Regional and/or Non-Regional Node Stations According to Tumor Location

The overall incidence of LN metastasis in the regional LN only and regional with non-regional LN was 10.5% (25/237) [Table 4]. Additionally, 9.2% of positive tumors were confirmed in the regional LN stations only (22/237). Tumors located in the middle third and lesser curvature of the stomach were found to metastasize to LNs in both the regional and non-regional LN stations (6.0%). In six cases, metastases were only observed in non-regional LNs (2.5%). One patient whose tumor was located in the upper third and greater curvature of the stomach metastasized to #3, two patients with middle third and greater curvature tumors metastasized to #3, one patient with a middle third and posterior wall-side tumor metastasized to #7, and one patient with a lower third and lesser curvature tumor metastasized to #5. In one patient whose tumor was in the lower third and greater curvature side, LN recurrence was confirmed in LNs #6, 7, and 11p after SNNS.

**Table 4:** Distribution of lymph node metastases to regional and/or non-regional node stations according to tumor location

	Upper-LC [*n* = 3]	Upper-GC [*n* = 5]	Upper-AP [*n* = 5]	Middle-LC [*n* = 50]	Middle-GC [*n* = 46]	Middle-AP [*n* = 45]	Lower-LC [*n* = 26]	Lower-GC [*n* = 28]	Lower-AP [*n* = 29]
Regional node stations (−)	3 (100)	4 (80.0)	4 (80.0)	44 (88.0)	39 (84.8)	40 (88.9)	24 (92.3)	23 (82.1)	25 (86.2)
Regional node stations (+) only	0 (0)	0 (0)	1 (20.0)	3 (6.0)	5 (10.9)	4 (8.9)	1 (3.8)	4 (14.3)	4 (13.8)
Regional and non-regional node stations (+)	0 (0)	0 (0)	0 (0)	3 (6.0)	0 (0)	0 (0)	0 (0)	0 (0)	0 (0)
Non-regional node stations (+) only	0 (0)	1 (20.0)	0 (0)	0 (0)	2 (4.3)	1 (2.2)	1 (3.8)	1 (3.6)	0 (0)

Data are expressed as *n* (%)

LC lesser curvature, GC greater curvature, AP anterior and posterior

### Diagnostic Parameters of Sentinel Node Navigation versus Regional Lymphadenectomy

The sensitivity rates of sentinel basin dissection and regional lymphadenectomy to detect LN metastases were 96.8% and 80.6%, respectively, with a statistically significant difference (*p* = 0.016) [Table 5]. The PPV of sentinel basin dissection and regional lymphadenectomy was 6.7% and 12.0%, respectively (*p* = 0.201), while the NPV of sentinel basin dissection was significantly higher than that of regional lymphadenectomy (99.5% vs. 97.2%, *p* = 0.021)

**Table 5:** Comparison of the diagnosis of lymph node metastasis by sentinel basin dissection vs. regional lymphadenectomy

	Sensitivity (95% CI)	*p* value	False negative rate (95% CI)	*p* value	Specificity (95% CI)	*p* value	Positive predictive value (95% CI)	*p* value	Negative predictive value (95% CI)	*p* value
Sentinel node navigation surgery	96.8% (88.9–100.0)	0.016	3.2% (0.0–11.1)	0.016	100% (100–100)	> 0.999	6.7% (0.0–16.7)	0.201	99.5% (98.5–100.0)	0.021
Regional lymphadenectomy	80.6% (65.5–93.1)		19.4% (6.9–34.5)		100% (100–100)		12.0% (0.0–26.9)		97.2% (94.7–99.1)	

CI confidence interval

## Discussion

This study proposed the concept of regional lymphadenectomy omitting SNN, and analyzed its diagnostic accuracy. The predefined criteria for regional lymphadenectomy—targeting LNs nearest to the tumor and in high-risk metastatic zones—were less effective in identifying metastatic nodes than those for SNN. Notably, the clinical possibility of regional lymphadenectomy was compared with that of SNN using a prospective, multicenter, randomized controlled trial.

EGC treatment is a continually evolving field, tending towards treatments that balance oncological safety with a patient’s quality of life. Optimal surgical approaches for EGC depend on an intricate understanding of its lymphatic spread. There have been no existing reports on regional lymphadenectomy regarding tumor locations. Kampschöer et al.^14^ developed the Maruyama computer program for a database of 3843 patients with gastric cancer who were treated by extensive lymphadenectomy. The Maruyama program comprised seven variables, including age, sex, gross type of tumor, presumed depth of primary tumor, tumor location, maximum tumor diameter, and histological type, and can predict the extent of LN dissection required for an individual patient. The accuracy of the Maruyama program was evaluated in several studies, and its high accuracy was proven to be a factor in the possibility of nodal metastasis and the prognostic factor of gastric cancer.^15,16^ However, the Maruyama database included both early and advanced gastric cancer, and the program showed lower accuracy in peri-gastric nodes than D2 +a (#13–16), and false positive rates.^17^ Moreover, it is not currently applied in clinical practice because it is complicated. Therefore, we defined the regional LN station for each tumor location in a simple and clinically applicable way.

Numerous studies of SNN have been performed in EGC cases. A meta-analysis of SNN examination with 46 studies, which included 2684 patients with gastric cancer, reported that the sensitivity, PPV, and NPV of SNN were 87.8%, 38.0%, and 91.8%, respectively.^6^ Another meta-analysis including 38 studies with 2128 patients demonstrated that the sensitivity and NPV of SNN were 93.7% and 76.9%, respectively.^18^ In that study, using data from the SENORITA trial, sensitivity, PPV, and NPV were 96.8%, 6.7%, and 99.5%, respectively, showing higher diagnostic parameters than previous analyses.

However, SNN requires the management and handling of isotopes, radiation hazards, and technical aspects, including intraoperative endoscopic injection of dual tracer and detection of the sentinel basins using a gamma probe. Even when performing SNN using fluorescence rather than isotope, special laparoscopic equipment is required. This surgical process requires a lot of manpower and longer operating time, which is not realistic regarding clinical practice in most medical centers. To replace the hassle of SNN, the concept of regional lymphadenectomy was introduced in this study. As previously explained, regional lymphadenectomy was defined as the LN area close to the location of each tumor and most likely to metastasize. In one retrospective study including 4929 patients who underwent gastrectomy for EGC, LN metastases were most frequently identified at station #3 (4.85%), followed by station #4 (3.05%).^19^ In a secondary analysis of the Japan Clinical Oncology Group study (JCOG0912), the relationship between tumor location and nodal station in patients who underwent distal gastrectomy for EGC was analyzed.^20^ In this study, nodal metastases were commonly found in #3 and #4d stations in the case of tumors located in the lesser and greater curvature sides, respectively. Additionally, anterior and posterior tumors frequently metastasized to #3 and #4d stations. As a result, our hypothesis of regional lymphadenectomy is theoretically consistent with the results of previous studies.

The proportion of metastasis to non-regional LNs was 2.5%, and the false negative rate of regional lymphadenectomy was 19.4%. The inadequacy of regional lymphadenectomy, as evidenced by its lower sensitivity and NPV, indicates a higher likelihood of missing metastatic nodes. These findings make it difficult to precisely pinpoint which LNs might be involved based on the tumor location.

Previous studies reported that larger tumor size and deeper invasion of tumor were associated with a higher false negative rate of sentinel node mapping.^21,22^ However, no significant factor was related to non-sentinel/non-regional LN metastasis in the present study. The contrary results might be due to lower proportions of patients with a large tumor, and pathologically T2 or higher in this study. In addition, the diagnostic values of SNN and regional lymphadenectomy including only patients with pathological T1 stage were further analyzed. The sensitivity and NPV of SNN were 96.0%, and 99.5%, respectively. Likewise, the sensitivity rate and NPV of regional lymphadenectomy were 84.0% and 98.0%, respectively. Even if only T1 cases were included, the predictive value of two procedures showed similar tendency.

Given this multidirectional lymphatic flow, the concept of regional lymphadenectomy for EGC seems clinically impossible. The variability and unpredictability in lymphatic spread patterns emphasize the need for sentinel node detection and dissection. By directly identifying the sentinel basins and assessing them for metastasis, SNN is a more precise method to determine the extent of lymphadenectomy, ensuring oncological safety.

To reduce the inconvenience of SNN using isotopes, the upcoming SENORITA IV trial is being conducted to evaluate the feasibility of laparoscopic SNNS using only fluorescence (NCT05978882). If no difference is found in the detection rate of sentinel basins using fluorescence compared with isotope and dye, performing laparoscopic SNNS using fluorescence can improve effectiveness without affecting the oncological safety.

This study had some limitations. First, although the study used data from a multicenter, prospective study, incidences of upper-third tumors were low. Second, the regional lymphadenectomy range is uncertain and depends solely on the surgeon’s decision. Additionally, this study analyzed a small number of patients with LN metastasis. The frequency of each LN station should be identified topographically in a large-scale, prospective study.

## Conclusion

While the diagnostic value of sentinel basin dissection was high, selective regional lymphadenectomy according to tumor location was found to be less effective in identifying metastatic nodes than that of SNN. Predicting the extent of lymphadenectomy by performing regional lymphadenectomy in EGC was impossible. Therefore, SNN should be performed in stomach-preserving surgery for EGC to reduce the extent of LN dissection.

## Supplementary Information

Below is the link to the electronic supplementary material.

Supplementary file1 (DOCX 20 KB)
