# Supplementary material for: Feasibility of Regional Lymphadenectomy for Stomach-Preserving Surgery in Early Gastric Cancer Omitting Sentinel Node Navigation: A Post Hoc Analysis of the SENORITA Trial
Source: Ann Surg Oncol. 2024 Jul 31;31(10):6939–46. doi: 10.1245/s10434-024-15950-1 (PMC11413058; doi:10.1245/s10434-024-15950-1)
Supplement: Supplementary file 1 — Supplementary file1 (DOCX 20 KB) [file 10434_2024_15950_MOESM1_ESM.docx]

**Supplementary Table 1. Distribution of sentinel basins and lymph node metastases at each station according to the tumor location**

| Station | Upper-LC | Upper-GC | Upper-AP | Middle-LC | Middle- GC | Middle- AP | Lower-LC | Lower-GC | Lower-AP |
| --- | --- | --- | --- | --- | --- | --- | --- | --- | --- |
| No. 1 |  |  |  |  |  |  |  |  |  |
| SB^*^ | 100% (3/3) | 80.0% (4/5) | 40.0% (2/5) | 2.0% (1/50) | NA | 8.9% (4/45) | NA | NA | NA |
| LNM | 0% (0/3) | 0% (0/4) | 0% (0/2) | 0% (0/1) | NA | 0% (0/4) | NA | NA | NA |
| No. 2 |  |  |  |  |  |  |  |  |  |
| SB | NA | NA | NA | NA | NA | NA | NA | NA | NA |
| LNM | NA | NA | NA | NA | NA | NA | NA | NA | NA |
| No. 3 |  |  |  |  |  |  |  |  |  |
| SB | 66.7% (2/3) | 60.0% (3/5) | 80.0% (4/5) | 98.0% (49/50) | 34.8% (16/46) | 80.0% (36/45) | 96.2% (25/26) | 17.9% (5/28) | 75.9% (22/29) |
| LNM | 0% (0/2) | 33.3% (1/3) | 25.0% (1/4) | 12.2% (6/49) | 6.2% (1/16) | 8.3% (3/36) | 4.0% (1/25) | 0% (0/5) | 13.6% (3/22) |
| No. 4sa |  |  |  |  |  |  |  |  |  |
| SB | NA | NA | NA | NA | 2.2% (1/46) | NA | NA | NA | NA |
| LNM | NA | NA | NA | NA | 0% (0/1) | NA | NA | NA | NA |
| No. 4sb |  |  |  |  |  |  |  |  |  |
| SB | NA | 60.0% (3/5) | 20.0% (1/5) | NA | 10.9% (5/46) | 4.4% (2/45) | 3.8% (1/26) | 3.6% (1/28) | 17.2% (5/29) |
| LNM | NA | 0% (0/3) | 0% (0/1) | NA | 20.0% (1/5) | 0% (0/2) | 0% (0/1) | 0% (0/1) | 0% (0/5) |
| No. 4d |  |  |  |  |  |  |  |  |  |
| SB | NA | NA | NA | 6.0% (3/50) | 89.1% (41/46) | 37.8% (17/45) | NA | 89.3% (25/28) | 65.5% (19/29) |
| LNM | NA | NA | NA | 0% (0/3) | 14.6% (6/41) | 11.8% (2/17) | NA | 16.0% (4/25) | 5.3% (1/19) |
| No. 5 |  |  |  |  |  |  |  |  |  |
| SB | NA | NA | NA | 4.0% (2/50) | NA | 2.2% (1/45) | 3.8% (1/26) | NA | NA |
| LNM | NA | NA | NA | 0% (0/2) | NA | 0% (0/1) | 100% (1/1) | NA | NA |
| No. 6 |  |  |  |  |  |  |  |  |  |
| SB | NA | NA | NA | NA | 6.5% (3/46) | 2.2% (1/45) | NA | 21.4% (6/28) | 6.9% (2/29) |
| LNM | NA | NA | NA | NA | 0% (0/3) | 0% (0/1) | NA | 16.7% (1/6) | 0% (0/2) |
| No. 7 |  |  |  |  |  |  |  |  |  |
| SB | NA | NA | 40.0% (2/5) | 24.0% (12/50) | NA | 22.2% (10/45) | 15.4% (4/26) | 3.6% (1/28) | 10.3% (3/29) |
| LNM | NA | NA | 0% (0/2) | 16.7% (2/12) | NA | 10.0% (1/10) | 0% (0/4) | 0% (0/1) | 0% (0/3) |
| No. 8a |  |  |  |  |  |  |  |  |  |
| SB | NA | NA | NA | 6.0% (3/50) | 2.2% (1/46) | NA | 3.8% (1/26) | 3.6% (1/28) | 3.4% (1/29) |
| LNM | NA | NA | NA | 0% (0/3) | 0% (0/1) | NA | 0% (0/1) | 0% (0/1) | 0% (0/1) |
| No. 9 |  |  |  |  |  |  |  |  |  |
| SB | NA | NA | NA | 2.0% (1/50) | NA | NA | NA | NA | NA |
| LNM | NA | NA | NA | 0% (0/1) | NA | NA | NA | NA | NA |

**^*^**included patients who underwent sentinel node navigation surgery (n=237; Upper-LC (n=3), Upper-GC (n=5), Upper-AP (n=5), Middle-LC (n=50), Middle- GC (n=46), Middle- AP (n=45), Lower-LC (n=26), Lower-GC (n=28), Lower-AP (n=29))

*SB*, sentinel basin; *LNM*, lymph node metastasis; *NA*, not available
